# Efficacy of Eribulin Plus Gemcitabine Combination in L-Sarcomas

**DOI:** 10.3390/ijms24010680

**Published:** 2022-12-30

**Authors:** María López-Álvarez, Cristina González-Aguilera, David S. Moura, Paloma Sánchez-Bustos, José L. Mondaza-Hernández, Marta Martín-Ruiz, Marta Renshaw, Rafael Ramos, Carolina Castilla, Elena Blanco-Alcaina, Nadia Hindi, Javier Martín-Broto

**Affiliations:** 1 Institute of Biomedicine of Sevilla, IBIS, Hospital Universitario Virgen del Rocío-HUVR, Consejo Superior de Investigaciones Científicas-CSIC, Universidad de Sevilla, 41013 Sevilla, Spain; 2 Centro Andaluz de Biología Molecular y Medicina Regenerativa (CABIMER), Universidad de Sevilla-CSIC, Universidad Pablo de Olavide, 41092 Sevilla, Spain; 3 Departamento de Biología Celular, Facultad de Biología, Universidad de Sevilla, 41013 Sevilla, Spain; 4 Instituto de Investigación Sanitaria Fundación Jiménez Diaz (IIS/FJD), 28015 Madrid, Spain; 5 Pathology Department, University Hospital Son Espases, 07120 Palma de Mallorca, Spain; 6 Nodo Biobanco Hospital Universitario Virgen del Rocío-Instituto de Biomedicina de Sevilla, Biobanco del SSPA, Unidad de Anatomía Patológica, Hospital Universitario Virgen del Rocío, 41013 Sevilla, Spain; 7 Centro de Investigación Biomédica en Red Cáncer (CIBERONC), Instituto de Salud Carlos III, 28029 Madrid, Spain; 8 Medical Oncology Department, University Hospital Fundación Jimenez Diaz, 28040 Madrid, Spain; 9 Medical Oncology Department, University Hospital General de Villalba, Collado Villalba, 28400 Madrid, Spain

**Keywords:** leiomyosarcoma, liposarcoma, eribulin, gemcitabine, soft-tissue sarcoma

## Abstract

Although the overall survival of advanced soft-tissue sarcoma (STS) patients has increased in recent years, the median progression-free survival is lower than 5 months, meaning that there is an unmet need in this population. Among second-line treatments for advanced STS, eribulin is an anti-microtubule agent that has been approved for liposarcoma. Here, we tested the combination of eribulin with gemcitabine in preclinical models of L-sarcoma. The effect in cell viability was measured by MTS and clonogenic assay. Cell cycle profiling was studied by flow cytometry, while apoptosis was measured by flow cytometry and Western blotting. The activity of eribulin plus gemcitabine was evaluated in in vivo patient-derived xenograft (PDX) models. In L-sarcoma cell lines, eribulin plus gemcitabine showed to be synergistic, increasing the number of hypodiploid events (increased subG1 population) and the accumulation of DNA damage. In in vivo PDX models of L-sarcomas, eribulin combined with gemcitabine was a viable scheme, delaying tumour growth after one cycle of treatment, being more effective in leiomyosarcoma. The combination of eribulin and gemcitabine was synergistic in L-sarcoma cultures and it showed to be active in in vivo studies. This combination deserves further exploration in the clinical context.

## 1. Introduction

Soft tissue sarcomas (STS) are a group of neoplasms with mesenchymal origin, representing 1% of all cancers in adults, with a crude incidence rate of about 5.6 cases per 100,000 inhabitants per year. STS are characterized by heterogeneous molecular aberrations, varying biology, and variable responses to treatment [1,2]. Despite efforts made in recent decades, the standard first-line systemic therapy for these tumours is still doxorubicin alone or in combination, generally with ifosfamide [3]. The increase in survival expectancy detected in the last decade for advanced STS is related, at least in part, with the emergence of new drugs in second and subsequent lines. Eribulin is a synthetic analogue of halichondrin B, a natural compound extracted from the marine sponge *Halichondria okadai,* and it has been approved for the treatment of metastatic breast cancer, and recently also for unresectable or metastatic liposarcoma (mLPS) patients who have received a prior anthracycline regimen [4,5]. In the randomized, open-label, multicentre, phase III clinical trial, Schöffski et al. reported that eribulin showed significantly longer overall survival (OS) with respect to dacarbazine, in a population based on advanced LPS and leiomyosarcoma (LMS) patients [6]. In a subsequent histological subgroup analysis of this trial, longer OS was restricted to patients with LPS subtypes, pleomorphic LPS being the one with the longest difference in OS (22.2 vs 6.7 months) [7]. In the case of the LMS group, both OS and progression-free survival (PFS) were comparable in patients treated with eribulin and dacarbazine [8]. Furthermore, a non-significant difference was found according to the primary anatomic site, eribulin being more effective in non-uterine LMS [8]. In any case, eribulin was shown to be active, inducing partial responses, even in uterine LMS and in different subtypes of LPS with relatively low toxicity [9]. The mechanism of action of eribulin is based on its ability to block microtubule polymerization without affecting its shortening phase, which is the case with other microtubule-targeted anticancer drugs such as taxanes and vinca alkaloids [10,11,12]. In turn, eribulin disrupts the mitotic spindle leading to cell cycle arrest at the G2/M phase [10]. In prostate and breast cancer cell lines, the mitotic arrest is irreversible and if prolonged in time leads to apoptosis [10,13]. Another interesting feature of eribulin is its ability to regulate vascular remodelling [14]. Eribulin inhibits pericyte- and endothelial-driven in vitro angiogenesis, reducing the number of capillary networks in co-cultures of pericytes and endothelial cells [15,16]. It also reduces the expression of angiogenesis-associated genes, including vascular endothelial growth factor (VEGF), as well as of genes involved in Wnt, Notch, and Ephrin signalling pathways and related to a mesenchymal phenotype [17]. In vivo, eribulin increases microvessel density, as observed in breast cancer and LMS xenograft models, causing tumour vascular remodelling and increasing tumour perfusion [17,18].

Gemcitabine [2′-deoxy-2′, 2′-difluorocytidine monohydrochloride (beta isomer); dFdC] is a deoxycytidine analogue used in the treatment of a large spectrum of tumours, including STS [19]. Gemcitabine, in its tri-phosphorylated form, acts as a competitive substrate of deoxycytidine triphosphate, being incorporated into DNA during replication, inhibiting its elongation and causing a solid G1 cell cycle arrest leading to cell death by apoptosis [20]. In the metastatic setting of STS, it is administrated as a single agent or in combination with docetaxel and dacarbazine, showing activity in LMS [21,22]. Additionally, several clinical studies exploring gemcitabine in combination with other cytotoxic drugs, including paclitaxel [23], sirolimus [24], and pazopanib [25], suggested synergistic activity and proved the usefulness of gemcitabine in STS treatment. Nevertheless, tumours develop mechanisms of chemoresistance, which may justify the limited therapeutic effect of gemcitabine. Thus, new strategies are urgently required to potentiate its activity in STS [19]. A promising strategy is the combination of gemcitabine with anti-neoplastic drugs that can increase tumour perfusion, facilitating its delivery and intratumoral accumulation.

This study aims to investigate the potential synergism of eribulin plus gemcitabine in L-sarcomas in vitro, assessing the mechanisms underlying this synergism and the translation to in vivo studies to determine the effectiveness and safety of this drug combination, as well as to analyse the potential benefit in the clinical setting.

## 2. Results

### 2.1. Eribulin and Gemcitabine Combination Produces a Synergistic Effect in Cell Viability

**Figure 1:**
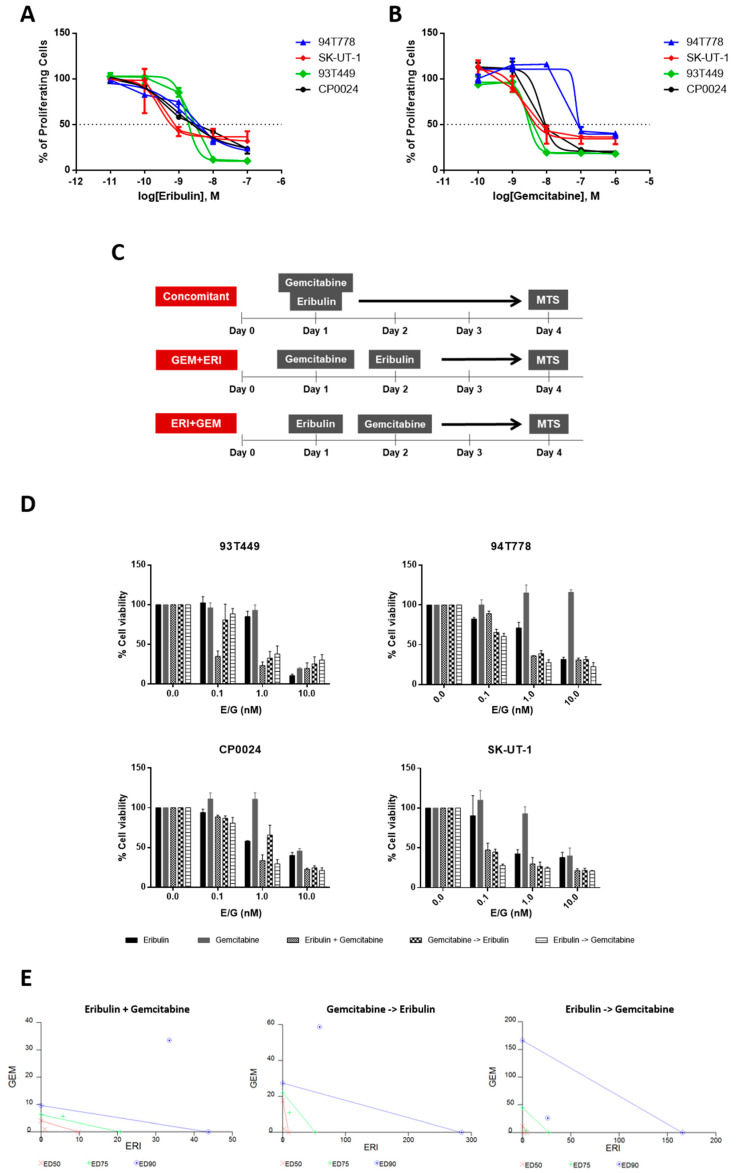
Cell viability after treatment with eribulin, gemcitabine or combinations in L−arcoma cell lines. (**A**) Cell viability measured at 72 h by MTS after treatment with eribulin at concentrations in the range of 10–11 to 10–7 molar or (**B**) gemcitabine at concentrations in the range of 10–10 to 10–6 molar in 94T778, SK-UT-1, 93T449 and CP0024. The graphs show the mean of 3 independent replicates performed in triplicate. (**C**) Representative diagrams of the different drug combinations tested in the cell lines. Cells were seeded on day zero and treated on day 1 and/or 2. Viability was measured on day 4 by MTS. (**D**) Cell viability in the LPS (upper graphs) and LMS (lower graphs) lines. The following conditions were tested: eribulin monotherapy, gemcitabine monotherapy, eribulin plus gemcitabine, gemcitabine pre-eribulin and eribulin pre-gemcitabine, all at 0.1, 1 and 10 nM concentrations of both drugs. The graphs show the mean of 3 independent replicates performed in triplicate (mean ± SD). (**E**) Isobolograms of the SK-UT-1 cell line showing the ED_50_ (red), ED_75_ (green), and ED_90_ (blue) for each of the combinations tested. SD: standard deviation; ERI: eribulin; GEM: gemcitabine; MTS: 3-(4,5-dimethylthiazol-2-yl)-5-(3-carboxymethoxyphenyl)-2-(4-sulfophenyl)-2H-tetrazolium, inner salt.

To look for more effective treatments for sarcomas, we tested combinations between eribulin and the cytotoxic agent gemcitabine in four sarcoma cell lines originating from LPS (93T449 and 94T778) and LMS (SK-UT-1 and CP0024). First, we identified the optimal drug concentration for each compound calculating the half-maximal inhibitory concentration (IC_50_) concerning cell viability in each cell line. MTS experiments analysed 72 h after adding the drug revealed IC_50_ viability values at nanomolar (nM) concentration in all the cell lines, confirming the cytotoxic effect previously described for both drugs (Figure 1A,B, Supplementary Table S1). Our results indicated that LMS cell lines were more sensitive to eribulin than LPS cell lines. Then, we tested whether the cytotoxic effect of eribulin could be potentiated by combination with gemcitabine by trying three different combinations: simultaneous addition of both drugs, sequential addition of eribulin followed by gemcitabine, and sequential addition of gemcitabine followed by eribulin. In all cases, viability was assessed by MTS after 72 h of the first drug treatment and in sequential regimens, the second drug was added 24 h after the first drug (Figure 1C). Drops in viability ranging from 90 ± 25.17-fold to 27.78 ± 2.52-fold in the SK-UT-1 cell line were observed when comparing monotherapy with eribulin and eribulin plus gemcitabine combinations (Figure 1D). This is especially apparent at low drug concentrations (0.1 and 1 nM) in all the cell lines. Looking at ED_50_ of the combinations, we observed that for both LMS cell lines, eribulin before gemcitabine is the most effective combination, with ED_50_ below 0.02 (0.199 for CP0024 and 6.272 × 10^−6^ for SK-UT-1 cell lines) (Figure 1D, Supplementary Table S2). Statistical analysis and isobolograms demonstrated that there was a synergistic effect in cytotoxicity when both drugs were combined, with the addition of eribulin before gemcitabine being the most effective combination and the one chosen for further analysis (Figure 1E and Supplementary Table S3).

Another way to look at viability is to perform a clonogenic assay that measures the ability of one cell to create a colony after drug treatment. In the 93T449 cell line, we observed that when cells were treated with the combination both at 12 h eribulin plus 6 h gemcitabine or 24 h eribulin plus 12 h gemcitabine, the ability of the culture to form clones was lower, with the difference with eribulin monotherapy being statistically significant (*p* = 0.002 and *p* = 0.033 for 12-6- and 24-12 h combination, respectively) (Supplementary Figure S1A–C). In the case of LMS cell lines, we observed a trend in the SK-UT-1 cell line both at 24 and 12 h that makes monotherapy have a lower clonogenic capacity than the combination (Supplementary Figure S1). The same effect is observed in the CP0024 cell line, with this difference being significant (*p* < 0.001 and *p* = 0.012 for 12 and 24 h of treatment, respectively). (Supplementary Figure S1).

### 2.2. Cell Viability Reduction in the Combined Treatment Is in Part Due to an Increase in Apoptotic Events

**Figure 2:**
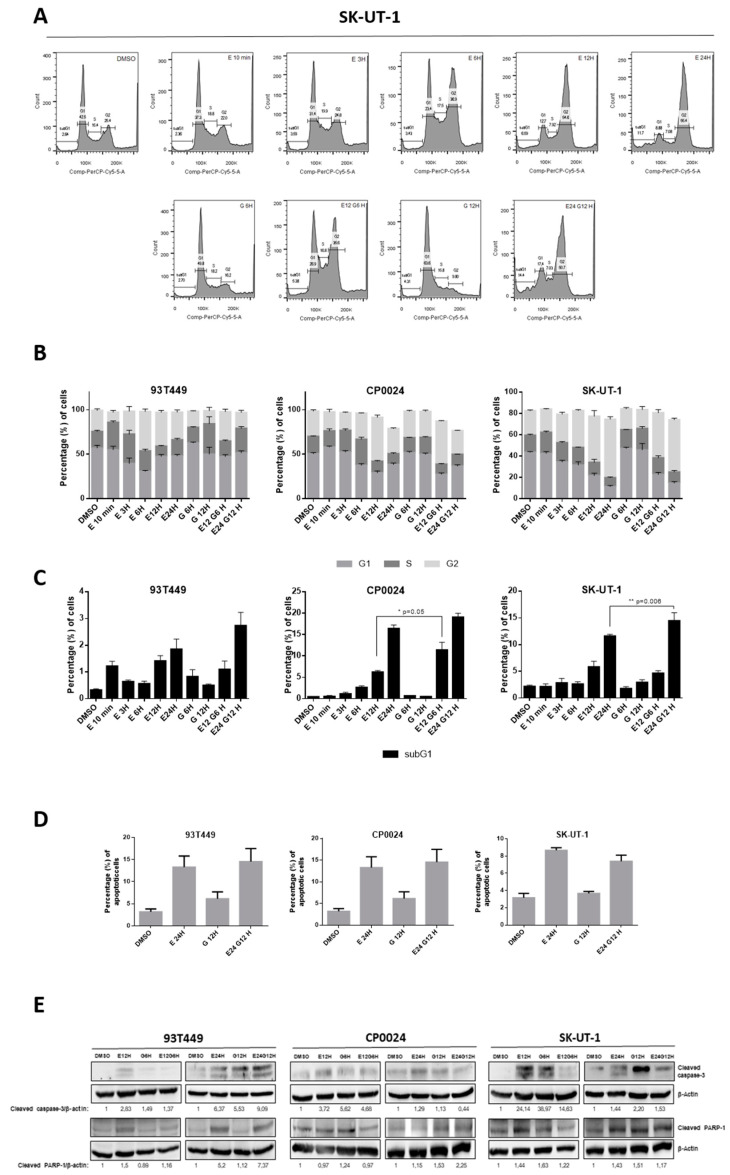
Study of changes in cell cycle and apoptosis after treatment with eribulin and gemcitabine. (**A**) Example of cell cycle profiles in the SK-UT-1 line after treatment with eribulin (1 nM) and gemcitabine (3 nM). From left to right; top row: DMSO, eribulin 10 min, 3, 6, 12 and 24 h; bottom row: gemcitabine 6 h, eribulin 12 h + gemcitabine 6 h, gemcitabine 12 h and eribulin 24 h + gemcitabine 12 h. (**B**) Percentage of cells in each cell cycle phase. The graphs show cells in G1, S, and G2 phase for 93T449, CP0024 and SK-UT-1 cell lines. 10 nM eribulin and 30 nM gemcitabine were used to treat 93T449 and CP0024 and SK-UT-1 were treated with 1 nM eribulin and 3 nM gemcitabine (mean ± SEM). (**C**) Percentage of cells in subG1 cell cycle phase (mean ± SEM) in 93T449, CP0024, and SK-UT-1 cell lines treated with the same schema as in B. (**D**) Cell apoptosis measured by flow cytometry after exposure to 10 nM eribulin and 30 nM gemcitabine in 93T449 and 1 nM eribulin and 3 nM gemcitabine in CP0024 and SK-UT-1. Eribulin was applied for 24 h and/or gemcitabine for 12 h and the graphs represent both early and late apoptotic cells (mean ± SEM). (**E**) Western-blot of the apoptotic markers fragmented caspase 3 and fragmented PARP-1, using antibodies against them and β-actin as a loading control. Drug concentrations were the same as in A and treatment times were 12 h of eribulin and/or 6 h of gemcitabine and 24 h of eribulin and/or 12 h of gemcitabine. Graphs show the mean of 3 independent replicates. SEM: Standard deviation from the mean. Student’s *t*-test for unpaired data: * *p* < 0.05; ** *p* < 0.005. SEM: Standard deviation of the mean.

To understand the molecular mechanisms responsible for the synergy observed in cell viability experiments with the sequential combination, we decided to check whether the reduction in cell viability could be a consequence of an increase in apoptotic events. Since, so far, all experiments have revealed similar results in the four cell lines tested, we decided to perform the subsequent analysis only in one LPS cell line (93T449) and two LMS cell lines (CP0024 and SK-UT-1); that became our focus of interest. Using flow cytometry to analyse DNA content, we quantified cell cycle profiles after the incubation with the drugs of interest. Consistent with the role of eribulin in microtubule polymerization, an arrest in G2/M is observed in long treatments and it seems to be reversible since the percentage of G2 cells diminished from 12 to 24h, except in the SK-UT-1 cell line (Figure 2A,B). However, interestingly, during short treatments with eribulin (from 10 min to 3 h) there was no accumulation of cells in G2/M but there was in S phase, suggesting that eribulin could also have an effect during DNA replication (Figure 2A,B). Additionally, we measured the hypodiploid events that represent the sub-G1 population, thus measuring DNA fragmentation occurring during apoptosis and cell death. Increasing incubation times with eribulin revealed that the longer the eribulin treatment was, the larger the sub-G1 population was, observed in 93T449, CP0024, and SK-UT-1 cell lines (Figure 2C). As in the MTS experiment, again we observed that LMS cell lines were more sensitive to eribulin showing higher levels of sub-G1 cells (17.45 ± 0.68% of cells after 24 h of treatment in the CP0024 and 11.6 ± 2.25% in the SK-UT-1 cell line) compared to the LPS 93T449 cell line (1.85 ± 0.21% after 24 h of treatment) (Figure 2C). This is true even considering that the eribulin concentration used in CP0024 and SK-UT-1 cultures was 10 times lower compared to the one used in 93T449 (1 nM vs. 10 nM). When we treated cells with eribulin followed by gemcitabine, we observed a slight increase in the number of sub-G1 cells with this difference being significant in both LMS cell lines (2.74 ± 0.49- E24hG12h vs. 1.85 ± 0.38-E24h in 93T449; 6.21 ± 0.4-E12hG6h vs. 11.39 ± 1.82-E12h in CP0024; 15.87 ± 0.74-E24hG12h vs. 11.6 ± 0.32-E24h in SK-UT-1) (Figure 2C). To test whether the increase in the sub-G1 population could be a consequence of an increase in the apoptotic events due to defects produced during the S phase or the arrest in G2/M upon eribulin treatment, we decided to measure apoptotic cells directly by flow cytometry with annexin V in both sarcoma cell lines. Figure 2D showed a clear increase in apoptotic events after treatment with eribulin for 24 h. However, no significant differences were observed between monotherapy and the combination with gemcitabine (Figure 2D). We also confirmed these results by measuring the levels of apoptotic markers, such as cleaved PARP-1 protein and cleaved caspase 3, by Western blot. We observed a significant increase in PARP and cleaved caspase 3 after 24 h of eribulin treatment compared to non-treated cells. However, the combination with gemcitabine did not increase the levels of these two proteins compared to eribulin in monotherapy (Figure 2E).

### 2.3. The Combined Treatment Produces an Increase in DNA Damage (γ-H2AX) and Accumulation of p21 Levels

**Figure 3:**
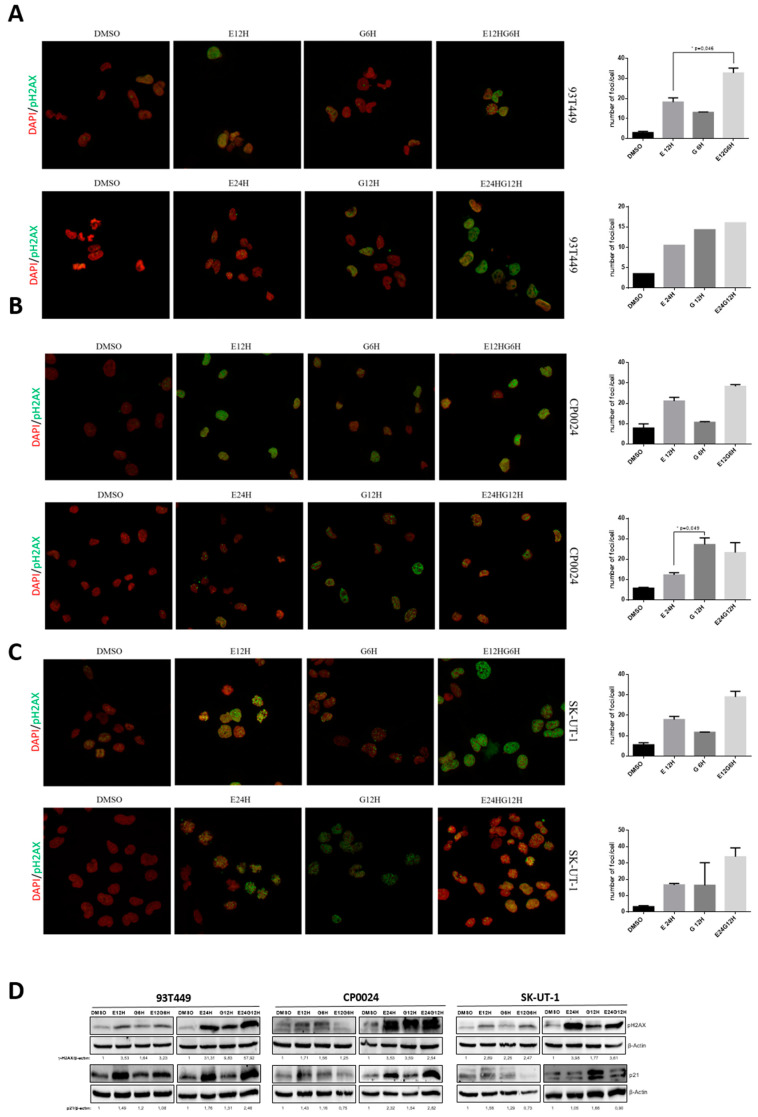
Study of DNA damage after combination of eribulin and gemcitabine in the 93T449, CP0024, and SK-UT-1 cell lines. (**A**) Immunofluorescence of γ-H2AX foci after 24 or 12 h treatment with eribulin (10 nM) and/or 12 or 6 h with gemcitabine (30 nM) in 93T449 cell line. Cells were stained with DAPI dye (nuclear control, red) and antibody against γ-H2AX (green) and quantification of the number of foci per cell. (**B**) Immunofluorescence of γ-H2AX foci after 24 or 12 h treatment with eribulin (1 nM) and/or 12 or 6 h with gemcitabine (3 nM) in the CP0024 cell line. Cells were stained with DAPI dye (nuclear control, red) and antibody against γ-H2AX (green) and quantification of the number of foci per cell. (**C**) Immunofluorescence of γ-H2AX foci after 24 or 12 h treatment with eribulin (1 nM) and/or 12 or 6 h with gemcitabine (3 nM) in SK-UT-1 cell line. Cells were stained with DAPI dye (nuclear control, red) and antibody against γ-H2AX (green) and quantification of the number of foci per cell. (**D**) Western-blot analysis of γ-H2AX and p21 protein levels after combination with eribulin and gemcitabine in 93T449, CP0024 and SK-UT-1 lines. Cells were treated in the same way as in (**A**–**C**). Antibodies against p21, γ-H2AX (ser139) and β-actin were used as loading control. Images were quantified with ImageLab. The graphs show the mean of 3 independent replicates and the SEM (standard error of the mean). Student’s *t*-test for unpaired data: * *p* < 0.05. E: Eribulin; G: Gemcitabine.

To assess the mechanisms implicated in the synergistic effect of the eribulin plus gemcitabine combination, we studied the accumulation of DNA damage by checking the presence of γ-H2AX, a marker of DNA damage accumulation in the cells. Interestingly, both in LPS and LMS cell lines, we observed an accumulation of γ-H2AX foci when cells were treated with eribulin and the combination both at 24 and 12 h (Figure 3). In the LPS cell line 93T449, the foci accumulation is greater with ERI + GEM than with eribulin alone, with this difference being statistically significant at 12 h (32.78 ± 2.40 vs. 18.24 ± 2.14; *p* = 0.046) (Figure 3A). The same difference is observed at protein level, especially for the 24–12 h experiment (Figure 3D). For the LMS cell line CP0024, we observe similar results in the 12-6H experiment, a foci accumulation when we treat both with eribulin or the combination (23.34 ± 4.74 in E12G6H vs. 12.33 ± 1.12 in E12H; *p* = 0.152) but in the 24-12H experiment we also observe an γ-H2AX foci accumulation when treating with gemcitabine that we did not observe in other cell lines, with a significant difference between G12H and E24H (27.19 ± 3.2 vs. 12.33 ± 1.12; *p* = 0.049) (Figure 3B). In the case of the LMS cell line SK-UT-1, we observe similar results to 93T449: an increment in foci accumulation and protein levels of γ-H2AX when we treat both with eribulin alone or in combination with gemcitabine. This accumulation is increased in the combination, and we observe a tendency that almost achieved statistical significance (29.05 ± 2.78 in E12G6H vs. 17.93 ± 1.57 in E12H; *p* = 0.073 and 33.9 ± 5.44 in E24G12H vs. 16.62 ± 0.85 in E24H; *p* = 0.088) (Figure 2C). Γ-H2AX protein levels are similar between combination and monotherapy with eribulin in the SK-UT-1 cell line (Figure 3D). Interestingly, while we observe a γ-H2AX accumulation, p21 protein levels were incremented with eribulin treatment, both in monotherapy or the combination in the 93T449 and CP0024 cell lines (Figure 3D).

### 2.4. The Combination of Eribulin and Gemcitabine Provides a Significant Benefit In Vivo Regarding Tumour Growth and Survival, Compared to Monotherapy

**Figure 4:**
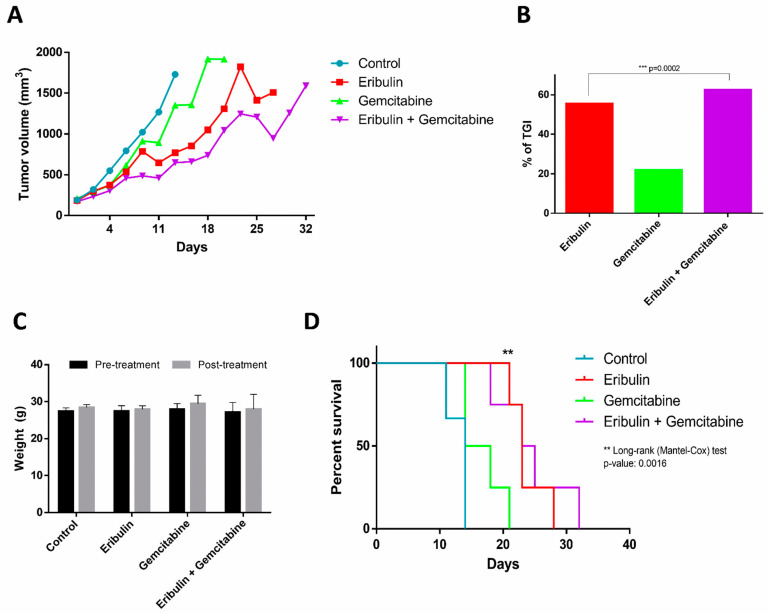
Effect of the combination of eribulin and gemcitabine in vivo in the PDX model LMS-IBiS-002. (**A**) Tumour volume, expressed in mm^3^, of mice subjected to each of the treatments: control (blue; n = 3), eribulin in monotherapy (red; n = 4), gemcitabine in monotherapy (green; n = 4) and eribulin + gemcitabine (purple; n = 4). Mice were sacrificed when they reached the volume of 1500 mm^3^. (**B**) Percentage inhibition of tumour growth with respect to the control group calculated on day 14 of treatment. Wilcoxon test for paired samples: *** *p* < 0.0005. (**C**) Weight, expressed in grams, of each experimental group before and after treatment with each of the drugs (**D**) Kaplan–Meier curve representing the survival probability of the different treatment groups. Log-rank test (Cox Mantel): ** *p* < 0.005.

**Figure 5:**
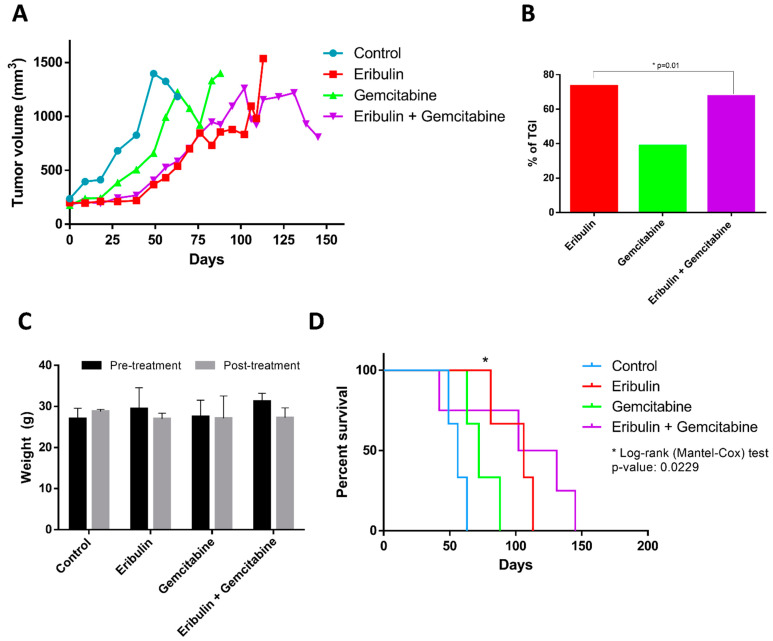
Effect of the combination of eribulin and gemcitabine in vivo in the PDX model LMS-IBiS-010. (**A**) Tumour volume, expressed in mm^3^, of mice subjected to each of the treatments: control (blue; n = 3), eribulin in monotherapy (red; n = 4), gemcitabine in monotherapy (green; n = 4) and eribulin + gemcitabine (purple; n = 4). Mice were sacrificed when they reached the volume of 1500 mm^3^. (**B**) Percentage inhibition of tumour growth with respect to the control group calculated on day 14 of treatment. Wilcoxon test for paired samples: * *p* < 0.05. (**C**) Weight, expressed in grams, of each experimental group before and after treatment with each of the drugs. (**D**) Kaplan–Meier curve representing the survival probability of the different treatment groups. Log-rank test (Cox Mantel): * *p* < 0.05.

To evaluate the in vivo relevance of eribulin and gemcitabine combination activity in tumour growth, we generated two LMS PDX mouse models (LMS-IBIS-002 and LMS-IBIS-010) and one LPS PDX model (LPS-IBIS-015). The original patient localization of tumour was uterine (LMS-IBIS-002) and deltoid (LMS-IBIS-010) LMS and retroperitoneal dedifferentiated LPS (LPS-IBIS-015) (Supplementary Table S4). We observed that at the beginning of the treatment both monotherapy with eribulin and the combined scheme suppress tumour growth, similarly, at later time points the combination regimen maintained a better suppression of tumour growth, while tumours treated only with eribulin persisted growing. This effect is observed in LMS-IBIS-002 (Figure 4A) and LMS-IBIS-010 (Figure 5A) models. The combination treatment group stayed alive for more days, whereas the eribulin monotherapy group had to be sacrificed earlier due to tumour growth. In both models, we also observed that eribulin alone or in combination with gemcitabine suppressed tumour growth compared to non-treated or gemcitabine-treated mice. In the LMS-IBIS-002 model, we calculated the percentage of tumour growth inhibition (TGI) to the control and on day 14 we observed that eribulin + gemcitabine TGI is statistically significantly greater than eribulin TGI (62.49 vs. 55.48%; *p* = 0.0002; Wilcoxon test (Figure 4B). We could not observe the same effect in the LMS-IBIS-010 model because differences between eribulin and the combination were reached when the control mice had already been sacrificed. At day 39, higher tumour growth inhibition was observed with eribulin, compared to the combination (73.5 vs. 67.59%; *p* = 0.01, Wilcoxon test) (Figure 5B). Bodyweight was not affected by any of the drugs administered (Figure 4C and Figure 5C). Both models showed a significant Kaplan–Meier curve between all groups (Log-rank (Mantel-Cox) test *p*-value: 0.0016 and 0.0229 for LMS-IBIS-002 (Figure 4D) and LMS-IBIS-010 (Figure 5D) models, respectively).

In the case of LPS PDX model LPS-IBIS-015, differences in tumour growth between the combination group and eribulin were also attained later than the 21-day cycle (Supplementary Figure S2A). TGI is also bigger in the eribulin group than in the combination at day 42 of the experiment, without reaching significance (81.65 vs. 67.87%; n.s.; Wilcoxon test) (Supplementary Figure S2B). Besides this, LPS-IBIS-015 presented a significant Kaplan–Meier curve between all groups (Log-rank (Mantel-Cox) test *p*-value: 0.0033) (Supplementary Figure S2D). Bodyweight is not altered by any of the treatments (Supplementary Figure S2C).

### 2.5. Eribulin Remodels Tumour Vascularity (IHQ CD31)

Based on the angiogenic capabilities of eribulin, we decided to study vessel formation in the tumours of our three PDX models in response to treatment (Figure 4E,F, Figure 5E,F and Supplementary Figure S2E,F). In the case of the LMS-IBiS-002 model, we did not observe significant differences between any of the treatment groups. In general, this model showed high cellularity but few microvessels (Figure S4E,F). In contrast, in the LMS-IBiS-010 model, the eribulin-treated group had the highest density of microvessels together with the combination, which was the second group with the highest MVD (Figure S4E,F). Finally, the LPS-IBiS-015 model showed the highest MVD (around 60 microvessels per field) but no differences were observed between any of the groups (Supplementary Figure S2E,F). The levels of apoptosis and necrosis were also evaluated in the tumours collected from the animals treated within this in vivo study by analysing the expression levels of apoptotic markers miRNA MIR184 and necrosis markers miRNA MIR21. No differences were seen in both miRNAs on the day the mice were sacrificed when comparing the expression levels of these markers in the mice treated with the combination group with mice treated with either eribulin or gemcitabine. Nonetheless, a tendency is observed for LMS-IBiS-010 model, where we observed that the combination presents higher levels of both miRNAs (Supplementary Figure S6).

## 3. Discussion

To identify a new strategy in the treatment of L-sarcomas in second or successive lines, we have focused on the combination of eribulin with gemcitabine. The combination is synergistic in in vitro experiments with LMS and LPS cell lines and it is particularly effective in PDX models of LMS. Eribulin is a microtubule-targeting agent that has been approved for the treatment of patients with unresectable or metastatic LPS previously treated with anthracyclines [5,26]. The use of eribulin for LMS has not been supported because, in a subgroup analysis, there was no apparent difference between eribulin and dacarbazine (standard active drug in this pathology) in OS (12.8 months in the eribulin arm and 12.3 months in the dacarbazine arm, respectively) and PFS (2.6 months in both treatment arms) [6]. In our study, we observed that the IC_50_ of eribulin for our cell lines is in the nanomolar range, as demonstrated by Hayasaka et al. [27], Stehle et al. [28], and by Escudero et al. in a panel of STS cell lines [29]. Our LMS cell lines are more sensitive to eribulin than LPS cell lines, contrary to what has been published in several clinical trials, so we are providing new data supporting the use of eribulin also for LMS. In Phase 3 clinical trial by Schoffski, the comparator used was not the most appropriate because leiomyosarcomas are a more sensitive subtype to dacarbazine than liposarcomas, thus compromising the outcome [6]. The greater sensitivity of LPS in the clinical setting may be because they have a more favourable microenvironment enriched in endothelial cells, which is a niche where eribulin can act more easily [15,30]. Since the approval of eribulin in STS, multiple clinical trials have been testing the safety and efficacy of eribulin in combinations with other drugs, such as pembrolizumab (NCT03899805), lenvatinib (NCT03526679) or irinotecan hydrochloride (NCT03245450), with no results published to date. In our study, we tested the combination of eribulin with gemcitabine. This combination has been tested in breast cancer: a Phase 1 trial was conducted in Japan in metastatic patients without reaching the recommended dose for Phase 2 due to haematological toxicities [31]. Additionally, a Korean group conducted a randomized Phase 2 trial with the combination of eribulin and gemcitabine (EG) versus paclitaxel and gemcitabine (PG) in first-line treatment of HER-2 negative patients, where it was observed that EG presents a similar clinical benefit to PG in terms of PFS, but with lower neurotoxicity [32]. For the doses used in that trial, they were based on a Phase 1 trial in advanced solid tumours [33]. A Phase 2 trial of the combination in L-sarcomas in Korea has been recently published showing promising activity of the combination with a good safety profile [34].

The combination of eribulin with gemcitabine was tested in our L-sarcoma lines with three different sequences: eribulin plus gemcitabine, eribulin before gemcitabine, and vice versa to find the best combination scheme to obtain a strong synergism. Eribulin before gemcitabine proves to be the best approach, as it is the approach with the lowest combination index (CI) in our cell lines. In the two LMS cell lines, CP0024 and SK-UT-1, the ED_50_ is lower when eribulin is applied before gemcitabine, and in the case of LPS, for 94T778 this is also the case but not for 93T449, where concomitance or gemcitabine before eribulin would work better. Regarding the combination indexes, there is a strong synergy in all cell lines when we treat with eribulin before gemcitabine at low concentrations (0.1 and 1 nM) of both drugs. This low concentration-synergy has been reported in other studies [35,36], probably due to high eribulin activity.

Previous preclinical studies described the G2 m arrest caused by eribulin, which becomes more pronounced over time, becoming irreversible [37]. Kuznetsov et al. thoroughly characterized the effect of eribulin on mitosis in lymphoma cell models. After eribulin treatment, cells begin to accumulate in the G2 m phase at 2 h, peaking at 12 h and hypodiploid events beginning to be observed thereafter. They also proved that these events were apoptotic by staining with acridine orange/ethidium bromide and by flow cytometry with annexin V [13]. In our case, we observed this blockade from 6 h of eribulin treatment in 93T449 and later in CP0024 and SK-UT-1, obtaining the maximum at 12 and 24 h, respectively, as previously described [37]. In addition, at short eribulin exposure, we observed an accumulation of cells in the S phase, suggesting that eribulin could have an additional effect during DNA replication not described previously. This effect is observable in 93T449 at 3 h and in CP0024 at 6 h. In parallel, an increase in the sub-G1 hypodiploid population is observed, as previously described in triple-negative breast cancer with the combination of eribulin and a histone deacetylase inhibitor [38]. Thus, there is an increase in cell death that is significantly different between eribulin and the combination in both CP0024 and SK-UT-1 that is not observed in the 93T449 cell line. This supports the idea that LMS, treated with a lower concentration of both drugs, is more sensitive to eribulin and that the combination potentiates an increase in cell death, observed by an increase in the sub-G1 population, and may be the cause of the observed synergy. It has been described that one of the effects caused by eribulin is an irreversible mitotic arrest, accompanied by Bcl-2 phosphorylation [10,13,39]. At the concentration and times used in our assays, eribulin does not appear to cause irreversible cell arrest in G2-M, although specific experiments, such as those named above, would be necessary to confirm this hypothesis.

Cell death occurs after mitotic arrest and is characterized by the inactivation of anti-apoptotic Bcl-2 proteins and by the activation of Bax in Ewing sarcoma cell lines, where caspases contribute only partially [40]. In rhabdomyosarcoma, Weiß et al. observed the need of mitotic arrest for apoptosis induction that it is not only caspase-dependent and ENDOG-silencing (caspase-independent pathway), it also decreases apoptosis [41]. Kuznetsov et al. also studied apoptotic markers, such as the activation of caspases 3 and 9 and PARP processing [13]. In our L-sarcoma lines, increased apoptosis was observed by both flow cytometry and PARP-1 and caspase-3 processing which would explain the synergy observed only in the LPS line. In our in vitro models of LMS, eribulin produces a greater increase in apoptosis than gemcitabine, but the combination of the two does not increase in a way that would be the sole cause of the synergy we observed when studying cell viability. This fact may be because other processes are involved in this synergy in addition to apoptosis or that apoptosis is not solely caspase-dependent. It is possible that by not observing a complete mitotic arrest, the cell is not fully entering apoptosis and alternative processes are occurring that lead to cell death. In the mice treated with eribulin combined with gemcitabine we did not observe an increase in the expression levels of markers of apoptosis and necrosis, which is in line with the results observed in the LMS in vitro studies. However, we need to take into account that these tumours were obtained after euthanizing mice a long time after their initial treatment with eribulin and/or gemcitabine, when these tumours had reached maximum tumour volumes. To perform a study of apoptosis and necrosis in tumour samples, we would need to have obtained tumours a few days after the initial treatment with the drugs, where we would probably have found significant differences in the expression of apoptosis/necrosis markers between treatment groups.

Gemcitabine, being a deoxycytidine triphosphate analogue, is incorporated into the DNA of cells during replication, causing DNA damage that the cell cannot repair, leading to G1-cell arrest. There is wide evidence of gemcitabine combinations with other drugs such as inhibitors of microtubules, PARP, or proteins such as p330. These induce a synergy caused by an increase in apoptosis, linked to an increase in DNA damage, which is measured, in most cases, by an increase in γ-H2AX [42,43,44]. These studies were conducted on both pancreatic and lung cancer, as there is not much evidence in STS. Likewise, gemcitabine alone produces an increase in γ-H2AX foci in in vitro models of pancreatic cancer [45]. In our LPS line, the combination of eribulin and gemcitabine causes an increase in the number of γ-H2AX foci per cell, which may justify the observed synergy. In the case of LMS, eribulin increases the number of foci, but there is no significant difference with the gemcitabine combination. The analysis of other markers of damage, such as Rad51 or the phosphorylation of ATM and ATR, would be necessary because different mechanisms of damage repair may be acting between the different lines [42,46]. DNA damage and other types of stress lead to increased expression of p21 [47]. In addition, some studies have co-localized p21 and γ-H2AX at the sites of damage since γ-H2AX is required for p21-induced cell cycle arrest to be induced following a replication error [48]. In our lines, p21 expression is induced in the same pattern as γ-H2AX after treatment with eribulin and gemcitabine.

Previous studies from our laboratory looked at the p53 status of our cells: 93T449 has no mutations, SK-UT-1 has two missense mutations at residues 524 and 743, and line CP0024 has a heterozygous mutation at residue 52. Recently, it has been shown that a complete loss of p53 function sensitizes lung cancer cells to eribulin [49]. This could be the case for our lines with mutation and loss of function of p53, more specifically in the LMS lines. The mutational status of 94T778 is unknown, but it would be of interest to determine whether *TP53* is mutated in this LPS line, which is more sensitive than the other line of this type of sarcoma (93T449) to eribulin, or to determine whether the expression/amplification levels of MDM2, a negative regulator of p53, are higher in 94T778. Both lines come from the same patient, but 94T778 comes from the second metastasis of that patient. In this case, the mutational/inactivation status of p53 could account for the different sensitivities observed in our preclinical setting. In line with this, it was recently reported that mutations in *TP53* are associated with a higher PFS in patients with LMS treated with eribulin [50].

In a phase-II trial measuring the efficacy of eribulin and gemcitabine combination in L-sarcomas, with PFS as the primary endpoint, treatment is carried out following the same scheme as in our in vivo PDX approach [27]. We were able to observe differences between eribulin and the combination group if, after one treatment cycle, we monitored tumour growth up to a maximum of 150 days in both LMS and LPS models. We found that tumours treated with the combination took longer to reach the maximum tumour volume than those treated with eribulin alone. Eribulin has shown antitumor activity both in monotherapy and in combination with various drugs in 10 xenografts of different types of cancer [14], and has shown superiority over other antitumor drugs in reversing doxorubicin resistance in an orthotropic dedifferentiated LPS xenograft [51] and in a Ewing’s sarcoma model [52].

It has been described that the antitumour activity of eribulin is also based on the changes it produces in the microenvironment, mainly through the associated immune response and vascular remodelling. In breast cancer xenografts, eribulin improves tumour perfusion through vascular remodelling based on an increase in the number of small functional microvessels [17]. In our case, in the LMS-IBiS-002 model, we obtained a tendency such as that of Miki et al. [53] in which treatment with eribulin reduces MVD. In contrast, in the LMS-IBiS-005 model, we obtained the expected result that is most reflected throughout the literature [14,17]: an increase in microvessels with treatment with both eribulin and the combination, i.e., eribulin would be causing an increase in tumour vessels, allowing greater perfusion of the drug and an enhancement of tumour growth. One of the reasons why we did not observe the expected results in MVD may be due to having performed this study at the end of the experiment. For a more reliable study of the changes in microvessel density, the tumours should have been removed one week after drug treatment and the study should have been performed at that time, which is one of the limitations of our study. Overall, the combination was found to be feasible and effective in LMS and LPS models, with no apparent toxicities, and with a delaying effect on tumour volume growth.

## 4. Materials and Methods

### 4.1. Cell Cultures

The LPS 93T449 (CRL-3043™), 94T778 (CRL-3044™), and LMS SK-UT-1(HTB-114™) human cell lines were obtained from the American Type Culture Collection (ATCC; Manassas, VA, USA). The CP0024 LMS human primary cell line was established from fresh tumour samples in our laboratory. The SK-UT-1 cell line was cultured in DMEM medium supplemented with 10% FBS, 1% penicillin/ampicillin (P/S), 1% sodium pyruvate, 0.1% MEM-non-essential amino acid solution, and 0.1% HEPES buffer. Both CP0024, 93T449, and 94T778 were cultured in RPMI medium supplemented with 10% FBS, 1% P/S, and 1% Fungizone. All cell lines were maintained at 37 °C with 5% CO_2_. Tissue culture supplements were all purchased from Sigma-Aldrich (Madrid, Spain). Cells were checked routinely and found to be free of contamination by mycoplasma or fungi, and their authenticity was checked before experiments. All the cell lines were discarded after 7–8 passes and new lines were obtained from frozen stocks.

### 4.2. Cell Treatments and IC_50_ Determinations

IC_50_ concentrations, which induced 50% cell death, were calculated for eribulin (kindly provided by Eisai Inc. (Tokyo, Japan)) and gemcitabine hydrochloride (Sigma Aldrich) in all cell lines. DMSO was used as a drug vehicle and negative control. SK-UT-1 (2 × 10^3^), CP0024 (2.5 × 10^3^), 93T449, and 94T778 (3 × 10^3^) cells were seeded in 96-well plates and treated for 72 h (h) with drug concentrations ranging from 10^−7^ to 10^−11^M for eribulin and from 10^−6^ to 10^−10^M for gemcitabine. Cell viability was measured using the 3-(4,5-dimethylthiazol-2-yl)-5-(3-carboxymethoxyphenyl)-2-(4-sulfophenyl)-2H-tetrazolium, inner salt (MTS) method (Promega; Madison, WI, USA), recording absorbance at 490 nm in an iMarkTM microplate absorbance reader (Bio-Rad, Hercules, CA, USA) spectrophotometer. Data were analysed using Prism 6.0 (GraphPad; San Diego, CA, USA).

### 4.3. Combination Treatment Assay

SK-UT-1 (2 × 10^3^), CP0024 (2.5 × 10^3^), 93T449, and 94T778 (3 × 10^3^) cells were seeded in 96-well plates, 24 h before treatment with eribulin plus gemcitabine (concomitant treatment), eribulin 24 h prior to gemcitabine and gemcitabine 24 h prior to eribulin in concentrations ranging 10^−8^ and 10^−10^ M for both drugs, always in a 1:1 ratio of both drugs. After 72 h, cell viability was measured using the MTS method (Promega; Madison, WI, USA), recording absorbance at 490 nm. The combination index ED50, ED75, and ED90 were then calculated with Calcusyn Version 2.0 (Biosoft^®^, Cambridge, UK), following Chou-Talalay’s combination index theorem.

For all remaining in vitro experiments, eribulin was tested at 10 nM (93T449) and 1 nM (CP0024 and SK-UT-1), and gemcitabine was used at 30 nM (93T449) and 3 nM (CP0024 and SK-UT-1) in the following conditions: eribulin monotherapy (24 or 12 h), gemcitabine monotherapy (24 or 12 h), and combination treatment (24 h eribulin plus 12 h gemcitabine or 12 h eribulin plus 6 h gemcitabine). DMSO was used as a drug vehicle and negative control.

### 4.4. Clonogenic Assay

Cells (3.5–4 × 10^4^) were seeded in 10 cm dishes and treated with the same scheme used in cell viability assays. Each condition was tested in triplicate. After 10 days, colonies were fixed and stained with crystal violet assay. After extensive washing, colonies were counted manually, and the relative number of observed colonies was represented in a graph.

### 4.5. Cell Cycle Analysis

After treatment, cells were trypsinized, centrifuged, and washed with 1X PBS, followed by a 30 min (min) incubation at 4 °C in 70% ethanol. After centrifugation at 1800 rpm for 10 min, cells were resuspended in a mixture of 1 mg/mL propidium iodide (Sigma Aldrich) and 50 mg/mL RNaseA (Qiagen^®^, Hilden, Germany) in PBS, incubating for 1 h at RT in the dark with horizontal shaking. The cell cycle was measured by flow cytometry (Canto II Analyzer cytometer (BD Biosciences; Franklin Lakes, NJ, USA)) and data were analysed with FlowJo software (FlowJo LLC; Ashland, OR, USA).

### 4.6. Apoptosis Analysis

The levels of apoptotic, early apoptotic, and necrotic cells were evaluated in the 93T449, CP0024, and SK-UT-1 cell lines. A FITC Annexin V Apoptosis Detection Kit with PI was used to determine cell death (Immunostep; Salamanca, Spain) following the manufacturer’s instructions. Apoptosis levels were determined by flow cytometry (Canto II flow cytometer) and data were analysed with both BD FACS Diva and FlowJo software.

### 4.7. Western Blotting

Cells were lysed in RIPA buffer (1M Tris-HCl pH 8 (PanReac AppliChem, ITW Reagents, Chicago, IL, USA), 0.5M EDTA (Thermo Fisher Scientific, Waltham, MA, USA), Triton™ X-100 (Sigma-Aldrich), 10% Sodium Deoxycholate (Sigma-Aldrich), 10% SDS (Sigma-Aldrich), and 3M NaCl (Thermo Fisher Scientific)). Equal amounts of total protein, determined by the Bradford method (Bio-Rad, Hercules, CA, USA) with QuickStart Bovine Serum Albumin Standard Set (Bio-Rad) for calibration curve, were separated by SDS-PAGE on 10–15% polyacrylamide gels and transferred to nitrocellulose membranes (Amersham™ Protran™ NC; GE Healthcare, Europe GmbH, Freiburg, Germany). For immunodetection, blots were soaked in 5% BSA (Albumin (BSA) Fraction V (pH 7.0) (PanReac AppliChem)) in TBS-T (1X TBS, 0.01% Tween^®^ 20 (Bio-Rad)) solution (blocking solution) and incubated with primary antibody in blocking solution overnight at 4 °C. The primary antibodies used were: β-actin (1:1000; A2103, Sigma-Aldrich), γ-H2AX (1:750; 9718, Cell Signalling), cleaved-caspase-3 (1:500; 9662, Cell Signalling), cleaved PARP-1 (1:750, 51-66396R, BD Biosciences), and p21 (1:1000, [EPR362] ab109520, Abcam, Cambridge, UK). Blots were then washed in 1X TBS-T and incubated with either rabbit anti-mouse IgG (1:10000; a9004; Sigma Aldrich) or goat anti-rabbit IgG (1:10000; ab6721, Abcam) peroxidase-labelled antibodies in 1X TBS-T for 1 h. HRP substrate was used for chemiluminescent detection (Amersham™ ECL™ Western Blotting Detection Reagent (GE Healthcare, Life Sciences)) and image acquiring was performed using Chemidoc Imaging System (Bio-Rad). Blots were analysed using Image Lab from Bio-Rad. The experiments were performed in triplicate.

### 4.8. Cell Immunofluorescence

Cells were seeded in 10 mm^3^ plates, where three sterile 1 cm circular coverslips had been previously introduced. After cell treatment, each crystal was transferred to a well of a 24-well plate, where the remainder of the protocol was carried out. Cells were fixed with 3% paraformaldehyde in H_2_O for 30 min at room temperature (RT). Then, cells were washed with 200 mM glycine solution for 15 min at RT and permeabilized with 0.5% Triton X-100 (Sigma-Aldrich) for 30 min at RT. Cells were blocked in 1% BSA 2X PBS solution for 30 min at RT. Subsequently, they were incubated with the γ-H2AX antibody 1:100 diluted in blocking solution overnight at 4 °C. Washes were made with 1X PBS for 5 min and incubated with the goat anti-rabbit IgG (H+L) Cross-Adsorbed Secondary Antibody, Alexa Fluor 488 (ThermoFisher Scientific) diluted in blocking solution for 1 h at RT in the dark. After washing with 1X PBS, nuclei were stained with DAPI (Life Technologies, Carlsbad, CA, USA) diluted in PBS (1:1000 dilution) for 15 min RT in the dark. Crystals were mounted on the slides with ProLong Gold solution (Life Technologies). The slides were stored at 4 °C until analysis. Images were obtained with the Leica TCS-SP2-AOBS confocal microscope and analysed with LCS Lite and Fiji Software v1.8.0.

### 4.9. Patient-Derived Xenograft (PDX) Models

Three PDX models were used for the in vivo studies: LMS-IBiS-002, LMS-IBiS-010, and LPS-IBiS-015. For LMS-IBiS-002, pathologic evaluation confirmed the original diagnostic of a leiomyosarcoma spindle cell type. Microscopically, it is a well-demarcated, unencapsulated tumour with an expansive growth front. It consists of interlacing bundles of spindle cells with eosinophilic cytoplasm. There were areas of intense nuclear pleomorphism, as well as frequent mitotic figures. No areas of coagulative necrosis, lymphatic vascular invasion were identified. Although, no immunohistochemical markers were stained for the original diagnosis, we were able to detect diffuse expression of smooth muscle actin (SMA) and h-caldesmon in our PDX. For LMS-IBiS-010, the patient tumour expressed SMA and h-caldesmon, while desmin protein expression was negative. Our PDX model maintained the positive expression of h-caldesmon, but had loss at least in the tumour block tested the expression of SMA. For LPS-IBiS-015, pathologist described the original tumour as a Grade 3 dedifferentiated liposarcoma with muscular differentiation, and this tumour was positive for MDM2, SMA, and Desmin and negative for myogenin, CD117, DOG1, and S100 protein. In our PDX samples, tumours maintained the positivity for SMA, MDM2, h-caldesmon, and DOG-1.

### 4.10. In Vivo Patient-Derived Xenograft (PDX) Studies

Six- to eight-week-old female nude mice (Nude-Foxn1 species (Charles River Laboratories, Wilmington, MA, USA)) were used. The mice were anesthetized with 100 µL of a mixture of the anaesthetics diazepam (Roche, Basel, Switzerland) and ketamine (Pfizer, New York, NY, USA) in a 1:3 ratio administered by intraperitoneal injection. Tumour samples (10 mm^3^ volume) were implanted subcutaneously on the right flank and developed in 3–7 weeks until reaching a minimum volume of 150 mm^3^ to start treatments. Tumours were measured using callipers.

### 4.11. In Vivo PDX Treatment

The mice were randomized according to their tumour size to the following 4 treatment groups: control group (intraperitoneal saline solution); eribulin group (1.6 mg/kg dose intravenously); gemcitabine group (120 mg/kg dose intraperitoneally); and eribulin and gemcitabine combination group, receiving doses of 1.6 mg/kg of intravenous eribulin and 120 mg/kg of intraperitoneal gemcitabine, eribulin 3 h prior to gemcitabine. For intraperitoneal treatments, we used an insulin needle (0.3 mm (29 G) × 12.7 mm) (BD Microfine; Beckton Dickinson and Company, Franklin Lakes, NJ, USA) and for intravenous treatment, we treated by either of the two lateral veins of the tail with a needle (0.40 mm (27G) × 10 mm) (BD Microfine; Beckton Dickinson and Company, USA). Mice were immobilized in a restrainer for mice of 35 g (90 × 30 mm) during tail treatment. The mice received the appropriate treatment for 2 weeks (1 dose/week, Day 0 and 7). Mice were monitored daily for signs of distress and weighed three times a week. The tumour size was measured, and size was estimated according to the following equation: tumour volume  =  [length × width^2^]/0.52, also three times a week. Mice were sacrificed when they reached the maximum tumour volume (1500 mm^3^) in a CO_2_ chamber, following the ethical standards of animal treatment. This project has the approval of Consejería de Agriculutra, Pesca y Desarrollo rural under the code 16/05/2017/061.

### 4.12. Immunohistochemical Analysis

Fragments of resected tumour PDX from nude mice were fixed in a 1:4 formol solution in H_2_O (Epredia™ Formal-Fixx™, Thermo Fisher Scientific) for 1 day and then paraffin-embedded. Cross-sections were prepared and stained with rabbit anti-mouse CD31, (PECAM-1) (D8V9E) XP^®^, Cell Signalling, 1:90 dilution) using Histofine Simple Stain Mouse MAX PO and DAB substrate kits (Nichirei Bioscience, Tokyo, Japan). Tissue morphology was visualized by haematoxylin and eosin (H&E) staining. Quantification of vascular morphology and microvessel density (MVD) was conducted by an expert pathologist (RR) with an Olympus BX61 optical microscope. To calculate the proportion (%) of small and large vessels, a piecewise threshold of the vascular area was selected. Vascular “hot spot” regions, defined as regions of high vascular density in the tumour, were identified at 100× magnification. Individual mature and immature microvessel counts were made on at least 5 different fields (0.95 mm^2^/field) and MVD was expressed as the average microvessel count per high-power field (HPF).

### 4.13. Apoptosis-Related miRNA Expression Analysis of Paraffin-Embedded Samples

Paraffin blocks corresponding to tumour samples from LMS-IBiS-002, LMS-IBiS-010, and LPS-IBiS-015 mouse models were cut into 10 µm slices. RNA, including small non-coding RNA, was extracted using the RecoverAll Total Nucleic Acid Isolation kit (Invitrogen, Waltham, MA, USA), following the manufacturer’s instructions. RNA samples were retrotranscribed to cDNA using the high-capacity reverse transcription kit (Invitrogen), following instructions from the cDNA TaqManTM Small RNA Assays user guide. TaqMan probes (Thermo-Fisher) for apoptosis miRNA MIR184 (Hs06637236_s1) and necrosis miRNA MIR21 (Hs04231424_s1) were used as targets, U6 snRNA (001973) was used as an endogenous control.

### 4.14. Statistical Analysis of In Vitro Studies

Data of biological replicates were grouped and presented as mean ± standard error of the mean. Differences between two treatment conditions were statistically analysed when indicated, using the unpaired Student’s *t*-test. Differences were considered significant when *p* < 0.05. Statistical analysis was performed using Prism 6.0 (GraphPad; San Diego, CA, USA). In addition, the log rank (Mantel-Cox) test was used for survival analysis in the PDX experiment, and the Wilcoxon signed-rank test was used for the assessment of tumour growth inhibition.

## 5. Conclusions

In conclusion, we have demonstrated that the eribulin and gemcitabine combination is synergistic in LMS and LPS cell lines. This synergism could be explained in part by the accumulation of DNA damage and the sub-G1 population. Eribulin plus gemcitabine combination is feasible in L-sarcoma PDX models, and survival in mice treated with the combination augments in comparison with eribulin monotherapy. Further analysis is needed to explain the mechanics underlying this synergism and its translation to the clinic onset.

## Data Availability

Not applicable.

## Supplementary Materials

The following supporting information can be downloaded at: https://www.mdpi.com/article/10.3390/ijms24010680/s1.
