# Supplementary material for: Efficacy of Eribulin Plus Gemcitabine Combination in L-Sarcomas
Source: Int J Mol Sci. 2022 Dec 30;24(1):680. doi: 10.3390/ijms24010680 (PMC9820645; doi:10.3390/ijms24010680)
Supplement: Supplementary file 1 [file ijms-24-00680-s001.zip › Supplementary Table S1.pdf]

Supplementary Table S1: IC_{50} values for liposarcoma and leiomyosarcoma cell lines.

	Eribulin (nM)	***Gemcitabine (nM)***
93T449	1,75	2,76
94T778	1,98	7,02
CP0024	0,95	6,81
SK-UT-1	0,28	2,10
