# Supplementary material for: Efficacy of Eribulin Plus Gemcitabine Combination in L-Sarcomas
Source: Int J Mol Sci. 2022 Dec 30;24(1):680. doi: 10.3390/ijms24010680 (PMC9820645; doi:10.3390/ijms24010680)
Supplement: Supplementary file 1 [file ijms-24-00680-s001.zip › Supplementary Table S2.pdf]

Supplementary Table S2: ED50, ED75 and ED90 in CP0024, SK-UT-1, 94T778, and 93T449 cell lines after different treatment sequences. ERI: eribulin; GEM: gemcitabine.

	ED50	ED75	ED90
**CP0024**			
Concomitant	0.336	1.184	4.250
GEM -> ERI	0.334	0.711	2.351
ERI -> GEM	0.199	0.244	0.316
**SK-UT-1**			
Concomitant	0.272	1.339	6.605
GEM -> ERI	0.023	0.337	9.236
ERI -> GEM	6.272e-006	0.138	18128
**94T778**			
Concomitant	0.894	0.700	0.761
GEM -> ERI	0.218	6.397	187.910
ERI -> GEM	0.168	0.579	2.001
**93T449**			
Concomitant	0.374	1.286	4.425
GEM -> ERI	0.377	0.6413	1.091
ERI -> GEM	0.628	0.671	0.800
