# Supplementary material for: Efficacy of Eribulin Plus Gemcitabine Combination in L-Sarcomas
Source: Int J Mol Sci. 2022 Dec 30;24(1):680. doi: 10.3390/ijms24010680 (PMC9820645; doi:10.3390/ijms24010680)
Supplement: Supplementary file 1 [file ijms-24-00680-s001.zip › Supplementary Table S3.pdf]

Supplementary Table S3: Combination indexes for the different sequencing combinations of eribulin and gemcitabine in cell lines CP0024, SK-UT-1, 93T449, and 94T778. The rows represent different concentrations of drugs in the rows and the different combination sequences in the columns. ERI: eribulin, GEM: gemcitabine, [E]: eribulin concentration, [G]: gemcitabine concentration.

CP0024

Sequence / [E] and [G] (nM)	Concomitant	GEM -> ERI	ERI -> GEM
**0.1**	0.096	0.207	0.274
**1**	0.252	0.355	0.083
**10**	1.964	0.636	0.429

SK-UT-1

Sequence / [E] and [G] (nM)	Concomitant	GEM -> ERI	ERI -> GEM
**0.1**	0.071	0.053	0.031
**1**	0.523	0.125	0.203
**10**	4.344	0.831	1.240

94T778

Sequence / [E] and [G] (nM)	Concomitant	GEM -> ERI	ERI -> GEM
**0.1**	1.115	0.046	0.174
**1**	0.290	0.380	0.176
**10**	2.513	3.583	1.124

93T449

Sequence / [E] and [G] (nM)	Concomitant	GEM -> ERI	ERI -> GEM
**0.1**	0.085	0.326	1.249
**1**	0.671	0.176	0.198
**10**	6.160	1.102	1.173
