# Supplementary material for: Efficacy of Eribulin Plus Gemcitabine Combination in L-Sarcomas
Source: Int J Mol Sci. 2022 Dec 30;24(1):680. doi: 10.3390/ijms24010680 (PMC9820645; doi:10.3390/ijms24010680)
Supplement: Supplementary file 1 [file ijms-24-00680-s001.zip › Supplementary Table S4.pdf]

Supplementary Table S4: Clinical and histopathological characteristics of patient-derived xenografts.

PDX MODEL	SEX	Age at diagnosis (Years)	Histological subtype	Histologic and molecular characteristics	Metastasis at diagnosis	Therapies pre-sample	Therapies post-sample	Status at last control
**LMS-IBiS-002**	Woman	53	Uterine LMS	High mitotic index<br>Spindle cell bundles<br>Nuclear pleomorphism	No	Surgery<br>GEM + DTIC<br>RT and Brachytherapy<br>Trabectedin	Olaratumab + DXR<br>Pazopanib	Dead
**LMS-IBiS-010**	Man	59	Deltoid LMS	SMA and Desmin positive<br>Caldesmon negative	Yes (lungs)	No	Olaratumab + DXR<br>Trabectedin + RT	Alive with disease
**LMS-IBiS-015**	Man	53	Dedifferentiated LPS	MDM2, SMA and desmin positive<br>Myogenin, CD117, DOG1 and S100 negative	No	No	Surgery<br>EPI + IFO	Dead

AML: smooth muscle actin; DOG1: discovered on GIST-1; DTIC: dacarbazine; DXR: doxorubicin; EPI: epirubicin; GEM - gemcitabine; IFO - ifosfamide; LMS - leiomyosarcoma; LPS - liposarcoma; MDM2 - murine double minute 2; RT – radiotherapy.
