# Supplementary figures and images for: Efficacy of Eribulin Plus Gemcitabine Combination in L-Sarcomas
Source: Int J Mol Sci. 2022 Dec 30;24(1):680. doi: 10.3390/ijms24010680 (PMC9820645; doi:10.3390/ijms24010680)

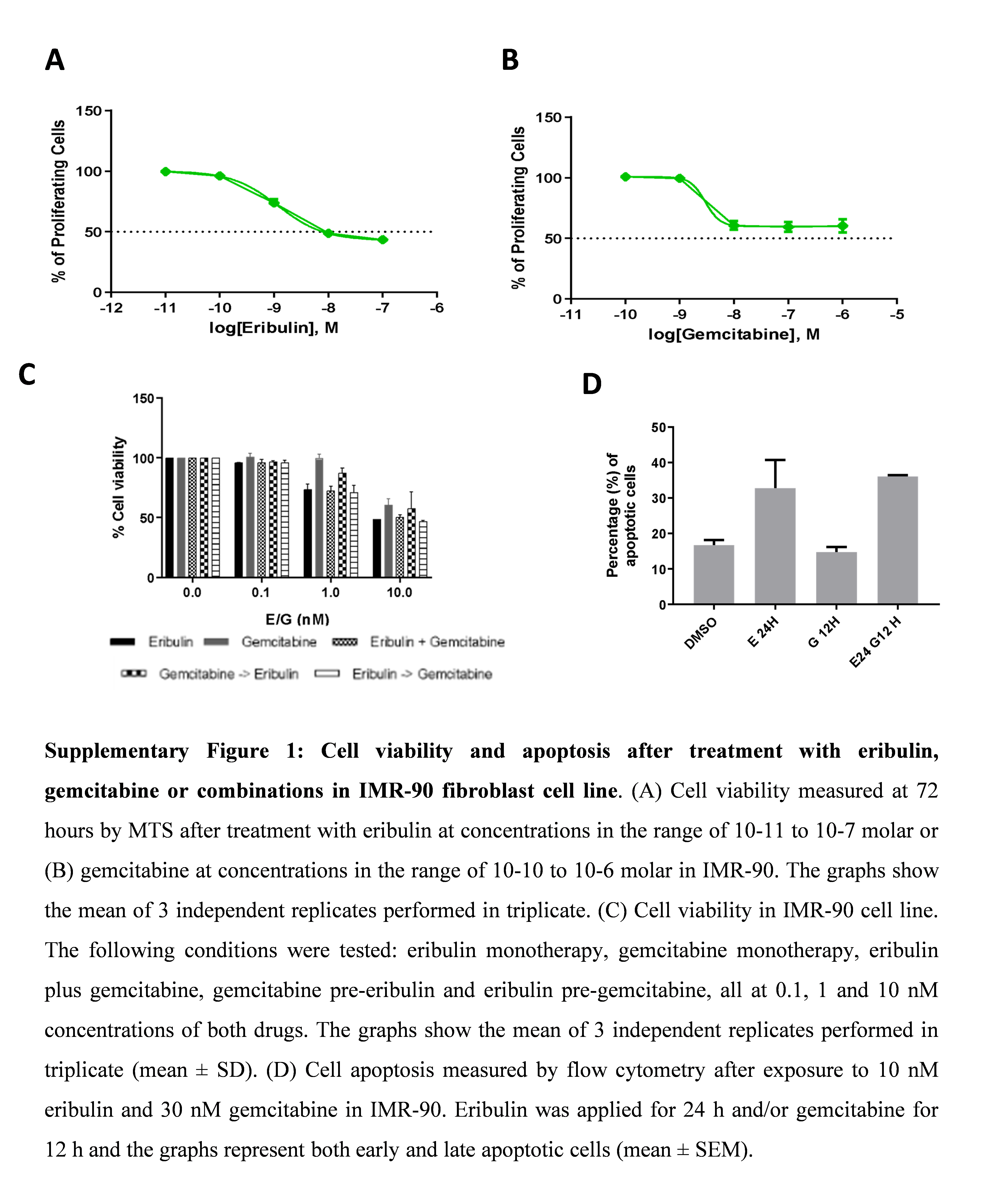

Supplement: Supplementary file 1 [file ijms-24-00680-s001.zip › Supp Figure S1.tif]

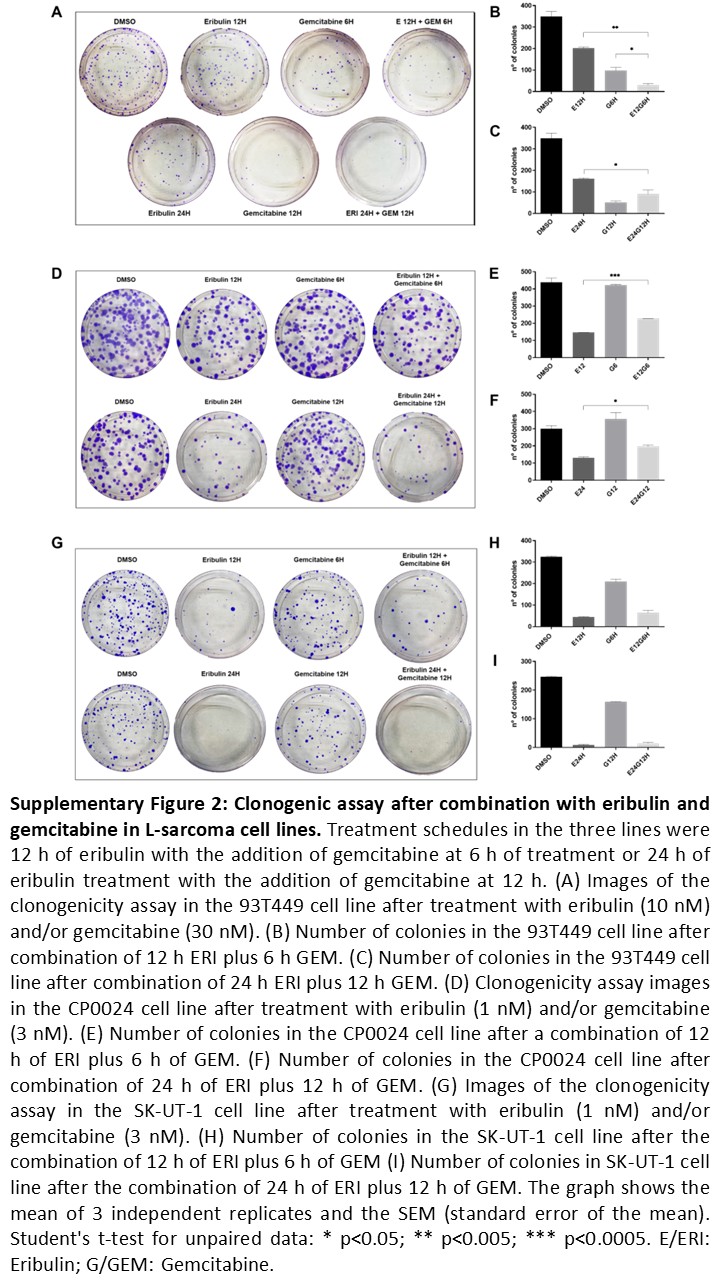

Supplement: Supplementary file 1 [file ijms-24-00680-s001.zip › Supp Figure S2.tif]

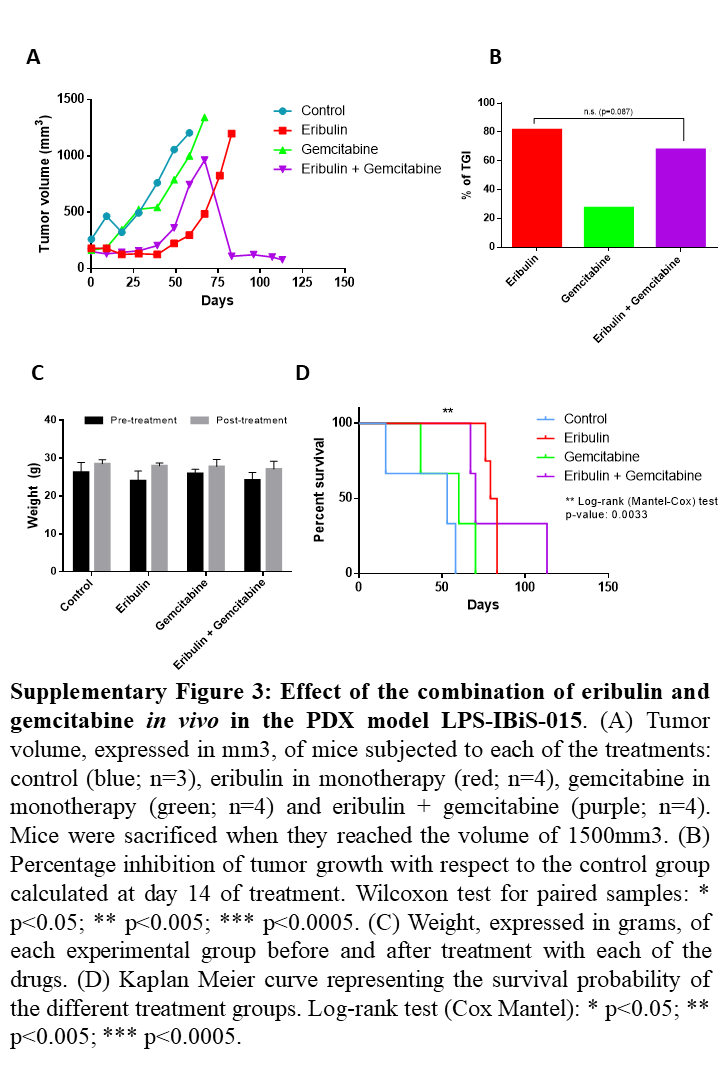

Supplement: Supplementary file 1 [file ijms-24-00680-s001.zip › Supp Figure S3.tif]

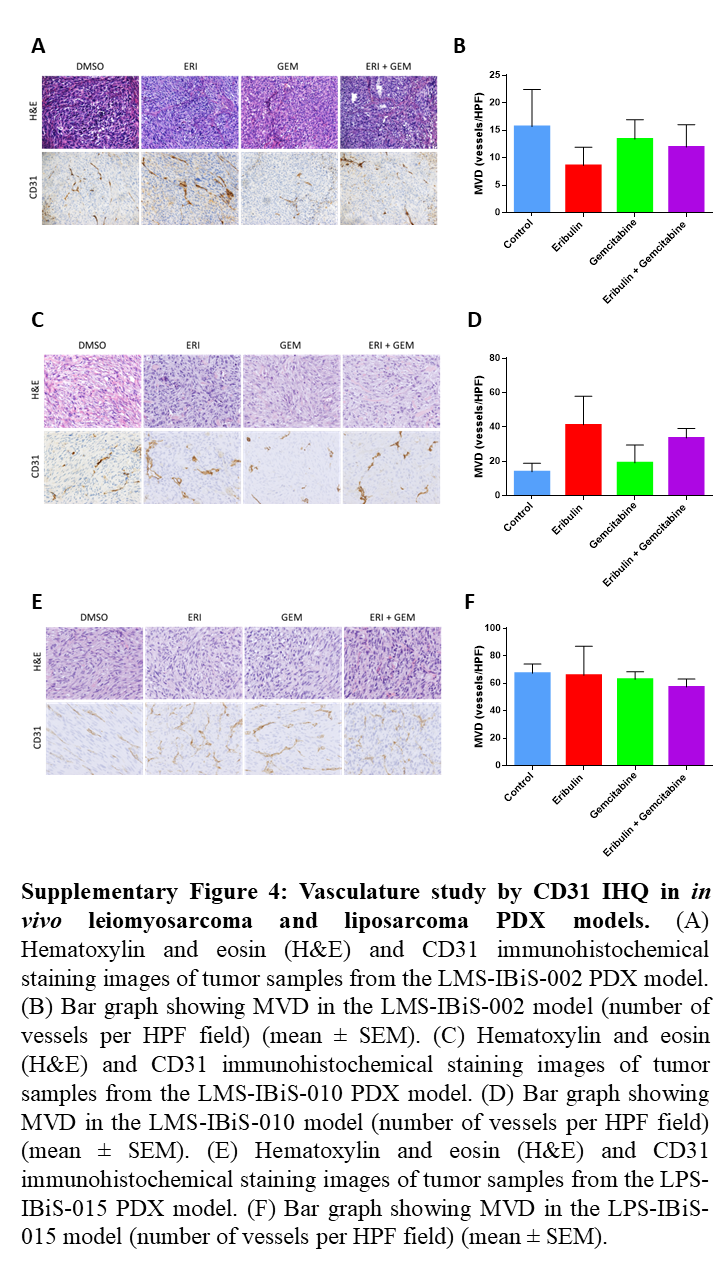

Supplement: Supplementary file 1 [file ijms-24-00680-s001.zip › Supp Figure S4.tif]

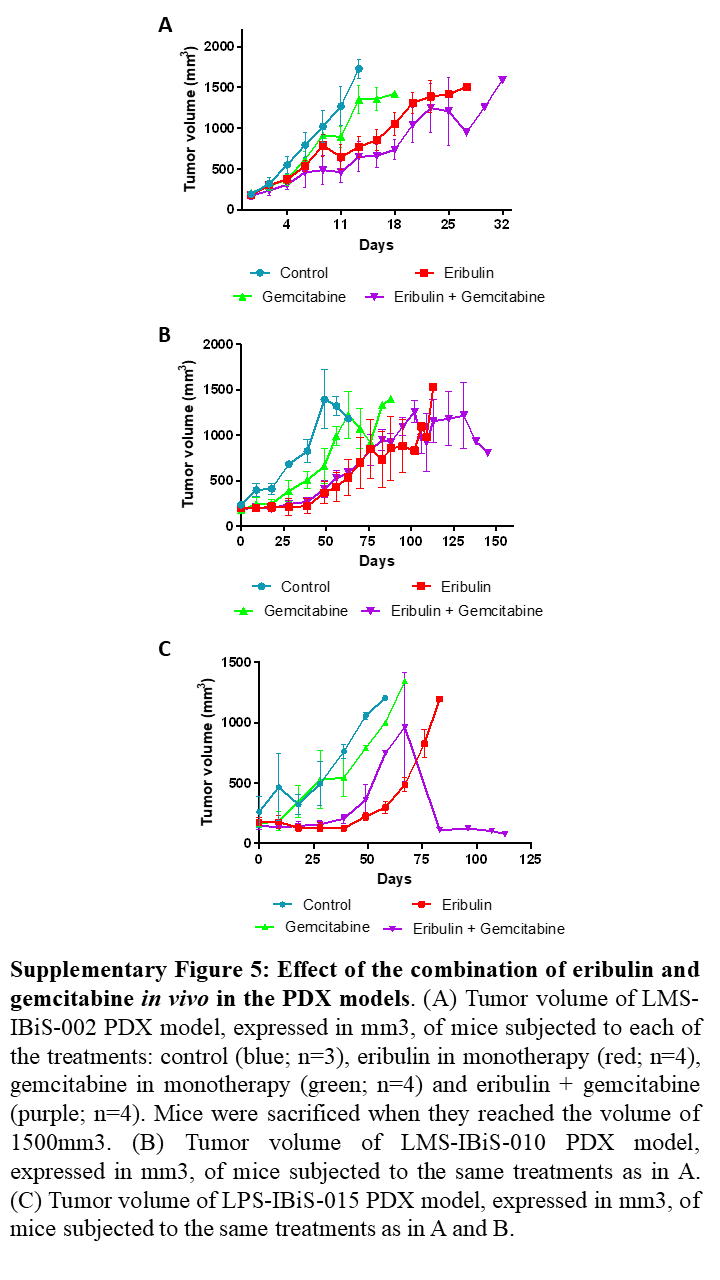

Supplement: Supplementary file 1 [file ijms-24-00680-s001.zip › Supp Figure S5.tif]

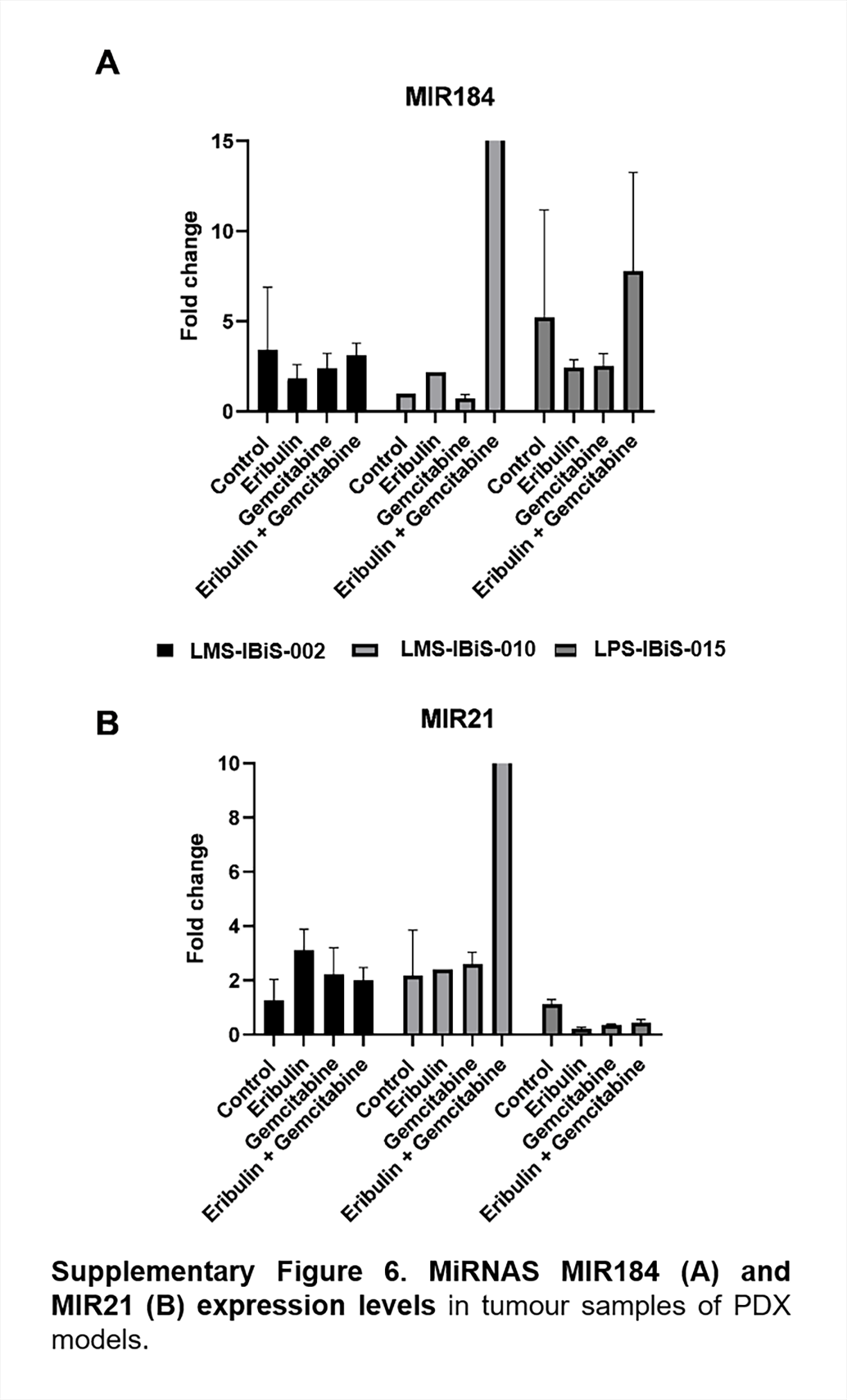

Supplement: Supplementary file 1 [file ijms-24-00680-s001.zip › Supp Figure S6.tif]
